# Supporting a Tsonga learner living with Bardet-Biedl syndrome, a rare complex disability

**DOI:** 10.4102/ajod.v12i0.1181

**Published:** 2023-12-04

**Authors:** Mfungana M. Shikwambana, Jean V. Fourie

**Affiliations:** 1 Department of Educational Psychology, Faculty of Education, University of Johannesburg, Johannesburg, South Africa

**Keywords:** adolescent education, Bardet-Biedl syndrome (BBS), progressive blindness, genetic disorder, polydactyly, psycho-educational support, special needs education, visual impairment

## Abstract

**Background:**

Bardet-Biedl syndrome (BBS) is a rare, systemic, hereditary disorder characterised by obesity, polydactyly, visual and auditory impairment, and cognitive disability. Providing quality education in appropriate schools for children who present with such complex chronic conditions is challenging.

**Objectives:**

This study explored the dimensions of psycho-educational support needs for a child with BBS in South Africa to contribute to the improvement of early detection and holistic interventions.

**Method:**

A descriptive in-depth qualitative case study of Gezani, an adolescent Tsonga boy diagnosed with BBS, was undertaken. Semi-structured interviews were conducted with his parents and teachers to ascertain the boy’s psycho-educational support needs. Medical reports provided information on the complexities and prognosis of the syndrome. Observations in the classroom corroborated the learner’s symptoms and behaviours.

**Results:**

Thematic content analysis revealed the key areas of support needs. Gezani’s cognitive disability required a modified, slow-paced curriculum. His visual impairment required mobility orientation training and learning Braille. His emotional needs were supported with psychotherapy to maintain a sense of well-being. Medical monitoring was recommended with interventions for walking and managing his diet and weight. Speech therapy supported his communication skills.

**Conclusion:**

Learners with multiple disabilities require carefully planned, individualised psycho-educational support programmes addressing their unique needs and delays with targeted remedial interventions in appropriate special needs schools.

**Contribution:**

This study informs educators about BBS and provides multi-faceted, holistic support. The Department of Basic Education could bring special schools and national policies in tighter alignment for learners presenting with complex disabilities.

## Introduction

As a school counsellor in a mainstream primary school, the researcher was introduced to Gezani when he was referred for a diagnostic psychological assessment. He was in grade six and was 14 years old at the time. He presented with multiple learning difficulties and unusual facial features and was overweight. He had been retained twice and thereafter progressed to higher grades on account of his poor academic performance. During the initial interview with his parents, we learned that Gezani had been referred to various specialists since his grade one year. These specialists included an occupational therapist, a speech therapist and a clinical psychologist who had recommended a special needs school. As no conclusive diagnosis was made, he remained in a mainstream school.

Gezani’s major challenge was his low vision as he struggled to read printed material and to see the chalkboard clearly. The author immediately referred Gezani to an optometrist. As his parents could not afford an optometrist, the researcher made an appointment for him at the University of Johannesburg Optometry Department where he was assessed free of charge. During the assessment, different lenses were tried to compensate for his low vision; however, instead of improving his vision, the lenses had the opposite effect, making his vision worse. The optometrists struggled to identify the cause of his visual impairment until one of them observed the scars on Gezani’s hands and asked if he had been born with six fingers. Gezani and his uncle who had accompanied him confirmed that this was the case. Gezani’s polydactyly was the first breakthrough in his diagnosis leading the optometrist to suggest that Gezani’s low vision might be related to a rare genetic disorder called Bardet-Biedl syndrome (BBS). An appointment with a geneticist was made, and a week later, the geneticist assessed him. The geneticist confirmed from clinical observations that Gezani had BBS. A blood test thereafter verified the diagnosis of BBS. After Gezani was diagnosed with BBS, the authors started researching the condition to learn more about it. The author wanted to know how BBS could be managed and how the support provided at school to Gezani could be improved.

## Bardet-Biedl syndrome

Bardet-Biedl syndrome is a rare, systemic, autosomal genetic disorder resulting from the disruption of cellular function (Ahmad & Noman 2022). The syndrome was named after George Bardet and Arthur Biedl who identified BBS in the early 1920s. This genetic condition is caused by anomalies in one of 19 genes that are named sequentially BBS1–BBS19. Among black South Africans, BBS is caused by the BBS10 gene (Fieggen et al. 2016; M’hamdi et al. 2011).

Bardet-Biedl syndrome presents with multiple clinical features that vary among diagnosed individuals. Visual impairment is the major symptom in all people with the condition and is because of progressive rod-cone retinal dystrophy in the eye (Forsythe & Beales 2013). Visual impairment ranges from partial visual loss to complete blindness, and eyesight deteriorates with age. Cognitive impairment ranges from moderate to severe intellectual disability (Castro-Sanchez et al. 2015). Children with BBS gain weight rapidly in their early childhood and tend to remain overweight during adolescence (Pomeroy et al. 2021). Individuals with Bardet-Biedl syndrome are often born with polydactyly (Forsythe & Gunay-Aygun 2020). Hypogenitalism is more frequent in men with BBS than in women with BBS (Tsegaw & Teshome 2021). Additional characteristics include impaired speech, dental abnormalities, partial or complete anosmia (sense of smell), and behavioural problems (Olson et al. 2019). The worst-case scenario is morbidity and premature mortality, caused by renal failure (Haws et al. 2016; Tobin & Beales 2007).

Although BBS is incurable and persists as a chronic condition, it is manageable, and most of the clinical features can be minimised with appropriate intervention and treatment focusing on the symptoms presented by individuals (Moore et al. 2012). Intervention measures given by medical geneticists are variably effective, depending on adherence and compliance. Geneticists recommend an annual renal ultrasound and renal function test as preventative measures because affected individuals are at risk of cystic renal dysfunction. Annual cholesterol and glucose tests and blood pressure measurement need to be done in view of the obesity associated with BBS. Consultation with a dietician is also vital to limit obesity-related complications. Annual hearing and eye tests are also needed as about half of the individuals diagnosed with BBS are at risk of developing hearing loss. Remedial therapies such as occupational, speech and physiotherapy should also be implemented (Tobin & Beales 2007).

## Bardet-Biedl syndrome and the African culture

People living with disabilities, along with their families, often face prejudice as beliefs and attitudes towards people with disabilities vary considerably between different cultures. Gezani and his family live in a township that comprises of African people from diverse cultures, which has resulted in him being subjected to the stigma and marginalisation associated with disabilities inherent in many African cultures. Parents of children with disabilities often keep them at home as a way of protecting them from such ostracism by the local community (Ndlovu 2016). Gezani’s parents tried to keep him hidden from the community, and his interaction with other children both at home and at school was limited.

Some African cultures adhere to the belief that disability is a punishment from angry ancestors (Ndlovu 2016). In many African cultures, people with disabilities are excluded from communities, and pressure is placed on families to seek intervention from traditional healers to correct the condition (Sait et al. 2011). Gezani’s family had consulted traditional Tsonga healers in Mozambique where rituals were performed to appease the ancestors in the hope that he would be healed of his disabilities.

These different challenges contributed to the family suffering emotional and financial stress in addition to them coming to terms with having a child diagnosed with the debilitating consequences of a condition such as BBS.

## Learners with Bardet-Biedl syndrome in school

As South Africa is committed to an inclusive education approach, all learners should be given quality education (Department of Education [DoE] 2001). As learners with BBS often manifest multiple impairments, including them in mainstream schools presents a challenge because of their high support needs. However, it is also challenging for special needs education to accommodate their complex needs. In South Africa, schools for learners with special educational needs are categorised as those for the visual and hearing impaired, the cognitively impaired and the physically impaired (DoE 2001). This categorisation poses complications when a learner manifests with physical, cognitive, visual and auditory impairments. Schools for physically impaired learners offer a mainstream curriculum. The provisions and adaptations at such schools are for mobility and access. On the contrary, in schools for the cognitively impaired, the curriculum is modified and adapted to suit the learner’s cognitive pace; however, these schools may not meet the physical and sensory support needs of the learner. A learner with multiple impairments will thus be disadvantaged when placed in any of these schools.

Previous studies on BBS focused only on the medical aspects, and there is limited research on how to provide psycho-educational support for learners with BBS during their school years. Psycho-educational support refers to all the support given to an individual or a group, with the primary goal of sharing information and helping the individual to develop better interpersonal and coping skills (Woolhouse, Copper & Pickard 2013). While researching the disorder, we realised that little research has been done on BBS, its impact on learning and how to support learners with BBS in schools. Because of the limited information available about this rare condition, we decided to conduct a case study to determine the psycho-educational needs of children with BBS.

## Aim

This study explored the different dimensions of psycho-educational support needed by a learner with BBS, through delving deeper into the difficulties and challenges faced. It is hoped that this information will contribute to the improvement of early detection and interventions for children with BBS and possibly other complex disabilities.

## Research methods and design

This case study used a qualitative research approach anchored in an interpretive paradigm which allows an in-depth understanding of a single person, their relationships and their environment (Thanh & Thanh 2015). This paradigm uses meaning-oriented methodologies such as interviews and observations (Creswell & Creswell 2022). A qualitative research approach seeks an in-depth, holistic understanding of people’s lived experiences within their natural environment (Flick 2018). This approach is flexible and responsive to changes that could occur during the study (Rahman 2017). This methodology thus made meaning of the lived experiences of Gezani and his family by interacting directly with them and providing a detailed account of their life world. To ensure the trustworthiness of the results of this qualitative study, we provided rich and detailed descriptions of the case (Creswell & Creswell 2022).

### Data collection and analysis

Semi-structured interviews were conducted focusing on Gezani’s psycho-educational support needs. Interviews were held with Gezani’s parents, his grade six class teacher and the school principal. An interview was conducted with a district official from the Gauteng Education Department’s support services. When Gezani was placed in a school for the visually impaired, his teacher at the new school was interviewed. These interviews were conducted in English which all participants understood, were audio-recorded and transcribed verbatim.

Various documents were collected including medical reports, a school report and printed emails of the communications between the researcher and the different professionals who were involved in the case. Medical reports were obtained from the optometrist, ophthalmologist, geneticist, physiotherapist, clinical psychologist and occupational therapist. Data were prepared by organising the interviews and the medical reports chronologically (Maguire & Delahunt 2017). The content of the data set was coded to extract patterns and themes using the six steps for thematic analysis as described by Braun and Clarke (2019).

### Ethical considerations

As this case study involved a highly vulnerable person, a minor child with a disability, careful planning and consideration was required in safeguarding the rights, dignity and well-being of all the participants. Informed, written consent was given by all participants. As a minor, Gezani gave assent. A pseudonym was used to protect the identity of the case participant and the family. Informed consent forms clearly explained the purpose, process and confidentiality of the study. Ethical clearance from the Faculty of Education Research Ethics committee at the University of Johannesburg was obtained (Ethical clearance number: 2016002). Approval was granted from the Gauteng Department of Education (DoE), as the study was conducted at a government school.

## Results

A brief history of Gezani’s case as derived from the participants and psychology reports is given first as context for the thematic analysis. Thereafter, Gezani’s psycho-educational support needs are presented as themes, with some reference to pertinent literature explaining unusual aspects of the raw data.

Gezani was born with polydactyly and brachydactyly, additional fingers and toes. His birth weight was within the normal range. He was born at full term by normal vaginal delivery, without any complications. He became ill at the age of 1 year and lost weight. He started gaining weight at the age of 3 years, after recovering from illness. He only started walking at the age of 18 months, and his speech development was delayed. His learning difficulties were first identified in grade one when he was referred for a psychological assessment. This assessment recommended he be placed in a special needs school, but instead he repeated grade one. Unfortunately, he remained in the mainstream school and later also repeated grade four. After the visual assessment and the BBS diagnosis in grade six, he was allocated a Learners with Special Educational Needs (LSEN) number for placement at a school for learners with visual impairment in grade seven.

Six themes were derived from the data analysis and are discussed below. The findings in each theme are supported by direct quotes made by the participants during the interviews and from the reports given by different healthcare professionals.

### Theme 1: Cognitive and intellectual support

According to his mother, Gezani presented with learning difficulties when he started school. His first school report stated: ‘He started grade one this year but is really struggling’. Cognitive impairment was confirmed by the psychological assessment done in his grade one year revealing that Gezani functioned within a borderline range of intellectual functioning. Another intellectual assessment was conducted when he was in grade six which indicated a mild intellectual impairment with an IQ score of between 70 and 80. A diagnosis of intellectual disability is consistent with the characteristics of BBS. Despite both these psychological reports diagnosing a cognitive impairment, he was not referred to or placed in a school for children with intellectual disabilities.

He had challenges expressing himself verbally because of his delayed speech development. Grade one assessments cover both oral and writing skills. Gezani did poorly in these grade-one assessments as he had difficulties with both receptive and expressive language. His mother reported that he could not speak properly, and she was concerned that he might have hearing problems. These delayed speech difficulties are consistent with BBS symptoms (Aravinda & Mohitha 2020).

Gezani’s academic performance continued to deteriorate each year, and he was retained in both the foundation and intermediate phases. The school principal confirmed, ‘This learner will not cope with a normal curriculum’. Because of his learning challenges, it was becoming more evident that Gezani was not coping at the mainstream school and needed to be placed in a school that would meet his special educational needs.

In grade seven, he was placed in a remedial class at a school for the visually impaired where he was taught general skills. The class was small with about 10 learners, which allowed the teacher to give each learner individual support and to monitor their progress more effectively. Observations showed that Gezani was benefiting from this class, compared to his previous classes where there were 50 learners, and the teachers were unable to give him attention or monitor his progress.

Children with BBS should be placed in special needs schools so that their cognitive impairment can be addressed effectively through early intervention and special education programmes sooner rather than later. These programmes designed for learners with BBS should be informed by psychological assessment results because children have different learning difficulties and, therefore, have different psycho-educational support needs.

### Theme 2: Visual impairment support

Visual impairment is one of the major features of BBS; therefore, tailored visual support is crucial in addressing individual psycho-educational support needs as the visual challenges differ from one learner to the other. People with BBS often have retinitis pigmentosa, which is a degeneration of the retina, causing blurred vision (Okoronkwo 2016). The symptoms usually appear during adolescence or young adulthood. In Gezani’s case, the symptoms were noticed in childhood when he started school. The severity of the condition differs from one person to another, and it cannot be treated. Some people can manage this condition by using glasses; however, Gezani’s condition is so severe that glasses would not help.

The Mohindra retinoscopy findings reported by the ophthalmologist also diagnosed Gezani with bilateral nystagmus, low vision and night blindness. The low vision affects Gezani’s reading and writing in class. He has challenges in seeing the written text and writing in his books. He often holds a book upside down without even realising that the text is upside down. He held the book extremely close to his eyes when he was reading and would lean close to the book when he was writing. He would blink often, and his eyes would move rapidly in different directions because of the nystagmus. Nystagmus is the involuntary back-and-forth movement of the eyes (Lemos & Strupp 2022). Usually, the rapid eye movement is side to side, but it can also be up and down or circular, as in Gezani’s case. The rapid movement happens in both eyes, at either a slower or faster rate. The symptoms of this condition include dizziness, difficulty seeing in the dark, holding the head in a tilted position and sensitivity to light. The optometrist noted that Gezani presents with all these symptoms.

Support for visual impairment should focus on the following areas: physical adjustment, curriculum adjustment, pedagogical practices and community support. Seating arrangements for visually impaired learners should be done according to the visual abilities and needs of each learner (Habulezi & Phasha 2012). Short-sighted learners usually sit in the front row, and far-sighted learners sit in the back row of the class. As Gezani is short-sighted, he should be seated in the front to have a better view of what is written on the board. Gezani should be given mobility orientation so that he can access the school environment with ease. As his vision is deteriorating and he is cognitively impaired, the mobility orientation should be done at regular intervals depending on how long it takes for him to master the layout of the school environment.

It was evident from the medical reports that Gezani will need intensive visual support: ‘I would recommend liaise with a school for the visually handicapped to assist him with the visual challenges’. His vision will deteriorate with time, resulting in permanent and complete blindness by late adolescence or early adulthood. This anticipated blindness requires Gezani to be trained in Braille and other functional skills. Braille is an effective tool for teaching literacy skills to visually impaired learners, and it serves as a life-long skill (Johnstone et al. 2009). However, Gezani was in a mainstream school for a long time and was only introduced to Braille when he was transferred to a school for the visually impaired in grade seven. Considering his age and cognitive impairment, it was going to be difficult for Gezani to learn Braille at this late stage and he required intense support.

Gezani required visual support for reading and writing. His reading books were in large print. He was given books with large thick lines for writing neatly and legibly on the page. His teacher gave this advice:

‘… we have a book with big lines … when he writes on that page he was able to write straight.’ (Teacher, School for Visually Impaired, Female)

‘And then for mathematics we made the large squares whereby when he writes the numbers he has to put numbers in each and every block until he comes better when it comes to writing.’ (Teacher, School for Visually Impaired, Female)

‘Enlarged print, he enjoys working with colours as he is not colour blind. In Gezani’s case, he has night blindness. We need the enlarged print for him.’ (Teacher, School for Visually Impaired, Female)

Considering his limited vision and difficulties with fine motor skills, Gezani was able to colour in drawings with clear bold outlines.

Assistive learning technology devices can be effective visual supports to address reading and writing difficulties. Gezani enjoyed using WhatsApp on his cell phone to text friends, family and his teacher. Even though he does not say much in his text because of his language barrier, he does have simple and short conversations and uses ‘emojis’ more than words. His teacher said he can benefit from using assistive technology devices:

‘[*B*]ecause I thought Gezani can be able to write maybe simple words and numbers to add numbers and whatever, but I was so surprised that Gezani was communicating with me using the cell phone WhatsApp.’ (Teacher, School for Visually Impaired, Female)

His teacher recommended that he learn computer skills ‘because academically he can’t cope with the mainstreams but maybe he can do some computers as time goes on’. As assistive devices could help learners with reading and writing, the occupational therapist noted, ‘This learner will not cope with normal teaching, and must use equipment to enhance his vision such as touch screens and text-to-speech for reading’. (Teacher, School for Visually Impaired, Female)

With appropriate visual support, Gezani can learn basic daily living skills and take part in various school activities. He had shown some improvement since he started at the school for the visually impaired. Teachers were doing their best to support him with writing, reading and acquiring functional living skills.

### Theme 3: Emotional support

The multiple disabilities associated with BBS can be the source of great emotional distress, and thus, counselling support was indicated. The support should seek to address the emotional challenges associated with the stigma of disability, visual impairment, cognitive impairment and obesity.

The stigma surrounding disabilities is present in Gezani’s community. His mother reported that the family ‘keeps to themselves’ and they are only now beginning to understand this disability as it affected her husband’s mother and Gezani’s younger sister as well. Gezani’s father is a carrier of the BBS gene but shows no symptoms.

Gezani is an introverted boy; he does not have any friends and seldom plays with other learners at school. The stigma is present at school as learners often shun learners in remedial classes. The teacher reported:

‘You know sometimes it’s difficult for learners in that class, because they are isolated from the mainstream, so, those who are in the mainstream they don’t usually associate themselves with those remedial ones.’ (Teacher, Mainstream school, Female)

His disability also contributes to his isolation. The teacher said, ‘But he just stays seated until I say, Gezani, its break time and then he said ok mam and then he went out, he has no friends’. (Teacher, Mainstream school, Female)

The teacher described a peer support group of learners at the school for the visually impaired called ‘soul buddies’, who are responsible for making friends with learners who might be isolated, new at the school or having challenges in making new friends. They spend time with these learners during breaks, orientating them about the school. As learners who are partially blind, the ‘soul buddies’ help those who are completely blind as they understand some of the emotions related to the difficulties of being visually impaired.

Learners with visual impairment usually have emotional challenges that are related to weather changes. Dark clouds affect their vision and they are unable to see clearly. When this happens, these learners tend to think that they are losing their vision which leads to heightened anxiety which they are unable to communicate to their friends and their teachers. The principal explained:

It’s a matter of mobility, when the weather has changed they sometimes get lost, they feel like they are losing their sight and it affects their behaviour in a way.’ (Principal, School for Visually Impaired, Male)

Gezani feels incompetent and sad as he struggles with his deteriorating sight. Emotional challenges are related to the ‘sight loss journey’ as people become more socially withdrawn and isolated (Habulezi & Phasha 2012). Supporting visually impaired learners requires the appreciation of the transitional processes and stages of visual loss deterioration, and therefore, specific counselling was indicated to address the emotional challenges associated with BBS.

### Theme 4: Medical support

Learners with BBS require regular medical check-ups to manage this chronic condition. It was evident from the geneticist report and interview that Gezani required regular medical support and assessments to manage his BBS.

Renal dysfunction is the main cause of death in BBS individuals and can develop at any time. Thus, it is crucial that renal functioning is closely monitored, and Gezani was advised to undergo annual renal assessments. The geneticist recommended:

‘Annual renal ultrasound and renal function tests - affected individuals are at risk of cystic renal disease and renal dysfunction. Annual cholesterol and glucose tests as well as blood pressure measurement in view of the obesity associated with the condition. Annual hearing test- 50% risk for hearing loss.’ (Geneticist, medical report, female)

The purpose of these assessments is to detect renal diseases at an early stage so that patients can start treatment immediately. The treatment is meant to combat secondary diseases linked to renal dysfunction, which can be life-threatening.

### Theme 5: Physical support

Learners with BBS often present with polydactyly. The most common intervention for polydactyly is the surgical removal of the extra digits on the hands and the feet. Most children have their extra digits removed within 2 years after birth (Welbourn et al. 2018).

This was the case with Gezani as reported by the geneticist, ‘Brachydactyly, as well as scars from the removal of postaxial additional digits’. His geneticist further stated, ‘the developmental delay is global, it involves gross motor and fine motor abilities’ (Geneticist, medical report). Gezani’s physical disabilities combined with his visual impairment impacted negatively on his mobility, resulting in poor motor co-ordination, execution and balance, and clumsy and slow movements. He was always accompanied to school by his uncle or mother, who held his hand to ensure that he did not bump into other people or obstacles in his way. He needed physical support regarding his mobility both at school and at home as his visual condition continues to deteriorate.

Gezani often complained about pain and discomfort in his feet related to the structure of his feet and the shape of the school shoes. He was more comfortable wearing sneakers as they are more flexible than school shoes and aid his balance and mobility. Because of BBS, Gezani is overweight which further impacts his mobility and other daily activities causing him to tire easily. The occupational therapist reported that Gezani was obese, weighing 78 kg when he was 14 years old. The normal weight for 14-year-old boys ranges between 47 kg and 51 kg (Forsythe & Beales 2013). Gezani was almost double the average weight for his age, and he was unable to take part in school sports. The occupational therapist reported:

‘On assessment, he has poor motor co-ordination, execution and balance, all movements are clumsy and slowed … he is unable to balance on one leg, hop, and skip and jump.’ (Occupational therapist, medical report, Female)

She recommended that a dietician be consulted to assist with a diet for controlling Gezani’s weight.

### Theme 6: Speech support

Gezani has challenges with communication, as he battles with comprehending language and expressing himself verbally. The occupational therapist reported ‘his speech is slurred and uncoordinated’. During their consultation with a clinical psychologist, his mother reported that Gezani could not speak to the doctor. When the authors first met Gezani, we also thought that he was unable to speak. He takes time to respond when spoken to and his voice is unclear and soft. It is challenging to hold long conversations with him because he responds with one-word answers. His teachers raised concerns about his communication challenges as this presented a barrier whenever there was something he needed to tell a teacher. For example, if he was being bullied, it was a challenge for him to report the bullying. He struggled with pronouncing the names of the bullies and was unable to explain the incident clearly to the teacher. His mother used to report the bullying on his behalf. His mother has a way of understanding what he is trying to communicate and is the only person with whom he is able to communicate effectively.

Both Gezani’s parents are Xitsonga speaking but they communicate with Gezani in isiZulu, which means he is not able to speak his mother tongue, Xitsonga. A person’s home language is not necessarily their dominant one but is rather the language that they use the most. Therefore, IsiZulu can be regarded as his main language, as it is the language that he is exposed to the most and the only language with which he communicates. The reason his parents communicate with him in isiZulu is that they live in a community that is predominately isiZulu speaking. Gezani attended an isiZulu kindergarten and takes isiZulu as his first language at school. His mother reported that this has presented a challenge when it comes to helping him with homework and language activities. Even though his parents can speak isiZulu, they are not proficient in reading and writing in isiZulu.

Gezani’s communication challenges increased when he had to learn English as an additional language at school because he had not yet fully mastered isiZulu as a home language. One’s home language is crucial in learning a second language (Liando & Tatipang 2022). It is much easier for children to learn a second language after mastering their home language. The home language is the basis for a child to learn a second language because he can refer to his home language when learning a new concept in the second language.

It is recommended that learners with speech impairments be referred to an audiologist, who will ascertain whether hearing loss might be a contributing factor. If there are difficulties with hearing, the auditory challenges should be addressed before any speech remediation can take place. It was recommended that speech therapy should be offered at the first signs of speech delays among children with BBS. Despite presenting with delayed speech, Gezani was only referred to an audiologist and speech therapist when he started school in grade one, but unfortunately, he never received any speech therapy. Although speech therapy is more effective when given at an early age (Aravinda & Mohitha 2020), late intervention could still be helpful in Gezani’s case.

## Discussion

Inclusive education is the practice by which the needs of diverse learners are successfully and adequately met (Robinson & Rusznyak 2020). Implementing quality inclusive education in South Africa has many challenges for children with complex disabilities, as special schools offer different curriculums and cater for different disabilities in their different categories. This categorisation is problematic for learners such as Gezani who presented with physical, visual and cognitive impairments. Furthermore, the curriculum offered at schools for children with physical disabilities follows the mainstream curriculum policy statement. The adaptations that are made at such schools are generally for mobility and accessing the school and learning resources only. On the contrary, in schools for the cognitively impaired, the curriculum is substantially modified and adapted. A learner who is both physically and cognitively challenged will therefore be disadvantaged when placed in either school. Learners with rare genetic disorders are likely to present with both cognitive and physical impairments and accommodating them in special schools is difficult. Bardet-Biedl syndrome is one such rare genetic disorder, and learners with BBS are less likely to have all their educational needs met as they present with multiple cognitive and physical problems. All these challenges require intensive support from various educational and medical professionals working in close multi-disciplinary teams and providing varied learning support strategies in the classroom (Yoro, Fourie & Van der Merwe 2020). With appropriate educational support, children with BBS can grow into adulthood having attained independent living skills (Forsythe & Gunay-Aygun 2020).

Gezani presented with multiple psycho-educational support needs in six domains, namely, cognitive, visual, emotional, medical, physical and speech. These support needs include remedial classes, counselling, learning assistive technologies, Braille training, buddies, regular medical check-ups, mobility orientation, regular physical exercise, dietary advice and speech therapy. Gezani’s support needs are based on his genetic condition, as well as cultural and societal beliefs and stereotypes. As Gezani and his family have been marginalised by the community where they live, which has had an influence on how he has been raised, family therapy is advised. To fully conceptualise Gezani’s psycho-educational support needs and those of other learners with BBS, multiple factors are considered related to the family background, the community, the school environment and educational resources as they all affect the individual and the psycho-educational support needs associated with BBS.

Even though his referral and placement at the school for the visually impaired were delayed for so many years, Gezani has shown steady improvement. The study suggests that had he been diagnosed sooner and been placed in an appropriate school from grade one, his learning difficulties would have been addressed earlier. The failure of the educational system to identify and provide appropriate support for Gezani highlights inadequacies in the implementation of Educational White Paper 6 (DoE 2001) towards an inclusive quality education system.

In summary, Gezani’s cognitive and intellectual challenges are addressed with an individualised support programme providing a modified and adapted curriculum. His visual impairment is managed with support at the school for visually impaired where he is taught orientation skills and reading in Braille that will help him when he becomes completely blind in early adulthood. Counselling therapy support assists Gezani and his family in dealing with emotional stresses and community stigma associated with BBS. Medical interventions related to the management of BBS’s potential renal complications should be scheduled annually. Support that will help Gezani with walking, balance and managing his weight is required from the occupational therapist and dietician. Speech therapy support assists him with communication skills and language pronunciation.

The rarity and complexity of BBS had an enormous impact on how this study was conducted. There was limited information about this genetic condition in the South African and global literature. Gezani is the first and only learner who has BBS at his school for the visually impaired. Teachers and officials in the Gauteng Education Department had never had a reported case of a learner with BBS and were therefore unable to provide the necessary psycho-educational support. The participants who were interviewed in this study had no prior knowledge about BBS. The teachers at his previous mainstream school and current school for the visually impaired and the officials at the district and head office in the Gauteng Department of Education (GDE) had the opportunity to learn about BBS and how to support learners with BBS through participating in this study.

The authors were personally involved in managing the case. The first author taught Gezani at his school and witnessed all his challenges in the classroom. The authors referred him to an optometrist with the permission of his parents and accompanied him and his uncle when he went for his visual assessment. They booked his genetic assessment appointment and accompanied him on the day of the assessment. His case was managed over a long period of time which gave the authors unique insights into the challenges faced by a person with a complex disability. At times, the authors visited him at the school for the visually impaired to see how he was coping there. The first author developed a close working relationship with his family, who were always available to discuss his progress. The authors knew that the transition from a mainstream school to a school for the visually impaired was a life-changing opportunity for Gezani to access appropriate psycho-educational support.

A limitation of this study is that it was a single-case study, and findings from this study might not be applicable to a larger group. The challenges that Gezani faces at school might differ from the challenges of another adolescent diagnosed with BBS, because of different family backgrounds, developmental milestones and life experiences. The findings from this case study could, however, be considered when giving psycho-educational support to children diagnosed with BBS or similar complex disabilities.

## Conclusion

This study explored the psycho-educational support needs of Gezani, an adolescent learner diagnosed with BBS who presents with multiple disabilities. Gezani experienced learning challenges from birth and struggled to cope with the mainstream curriculum at his previous school. Gezani is now in a school for the visually impaired, where his visual needs and learning difficulties are addressed. Teachers at the school for the visually impaired are learning about BBS and how to give Gezani appropriate psycho-educational support. Gezani is the first learner at the school to present with this rare genetic disorder; therefore, there is currently insufficient knowledge about BBS and support for Gezani at the school. The findings from this study, however, will inform the teachers about BBS and the psycho-educational support appropriate for Gezani.

Findings from this study can be used by the South African Department of Basic Education to improve its policies regarding learners with special educational needs. The rarity of BBS meant that teachers in this district were unaware that the condition existed, and therefore, they were unaware of the psycho-educational support needs of learners who are diagnosed with the condition. This study could help the Department of Basic Education to broaden its policy regarding inclusive education and special schools to include information and resources that will enable them to support learners with rare genetic conditions.

This case highlights multi-faceted interventions in supporting children with multiple disabilities. The findings show the challenges that other children with similar conditions may face. This case intervention was beneficial for Gezani, his family and immediate community, allowing them to understand this condition. The study could be beneficial for other adolescents diagnosed with complex visual, intellectual and physical disabilities, to receive quality psycho-educational support.
